# Discovery and Characterization of *Mycobacterium basiliense* sp. nov., a Nontuberculous Mycobacterium Isolated From Human Lungs

**DOI:** 10.3389/fmicb.2018.03184

**Published:** 2019-01-08

**Authors:** Helena M. B. Seth-Smith, Frank Imkamp, Florian Tagini, Aline Cuénod, Rico Hömke, Kathleen Jahn, Anne Tschacher, Peter Grendelmeier, Veronika Bättig, Stefan Erb, Miriam Reinhard, Gottfried Rütimann, Sonia Borrell, Sebastien Gagneux, Carlo Casanova, Sara Droz, Michael Osthoff, Michael Tamm, Ulrich Nübel, Gilbert Greub, Peter M. Keller, Adrian Egli

**Affiliations:** ^1^Division of Clinical Microbiology, University Hospital Basel, Basel, Switzerland; ^2^Applied Microbiology Research, Department of Biomedicine, University of Basel, Basel, Switzerland; ^3^Institute of Medical Microbiology, University of Zurich, Zurich, Switzerland; ^4^Institute of Microbiology, Lausanne University Hospital, University of Lausanne, Lausanne, Switzerland; ^5^National Center for Mycobacteria, Institute of Medical Microbiology, University of Zurich, Zurich, Switzerland; ^6^Division of Pneumology, University Hospital Basel, Basel, Switzerland; ^7^Division of Pneumology, Cantonal Hospital Baselland, Liestal, Switzerland; ^8^Division of Infectious Diseases and Hospital Epidemiology, University Hospital Basel, University of Basel, Basel, Switzerland; ^9^Swiss Tropical and Public Health Institute, Basel, Switzerland; ^10^University of Basel, Basel, Switzerland; ^11^Lungenpraxis Wohlen, Wohlen, Switzerland; ^12^Institute for Infectious Diseases, University of Bern, Bern, Switzerland; ^13^Department of Internal Medicine, University Hospital Basel, Basel, Switzerland; ^14^Leibniz Institute DSMZ, German Collection of Microorganisms and Cell Cultures, Braunschweig, Germany; ^15^German Center for Infection Research (DZIF), Braunschweig, Germany

**Keywords:** nontuberculous mycobacteria, novel species, *Mycobacterium basiliense*, virulence, pathogen

## Abstract

Bacteria belonging to the genus *Mycobacterium* are predominantly responsible for pulmonary diseases; most notably *Mycobacterium tuberculosis* causes granulomatous pulmonary infections. Here we describe a novel slow growing mycobacterial species isolated from respiratory samples from five patients, four with underlying pulmonary disease. The isolates were characterized by biochemical and molecular techniques, including whole genome sequencing. Biochemical characteristics generally match those of *M. marinum* and *M. ulcerans*; however, the most striking difference of the new species is its ability to grow at 37°C. The new species was found to grow in human macrophages, but not amoebae, suggesting a pathogenic rather than an environmental lifestyle. Phylogenetic analysis reveals a deep-rooting relationship to *M. marinum* and *M. ulcerans*. A complete genome sequence was obtained through combining short and long-read sequencing, providing a genome of 5.6 Mb. The genome appears to be highly intact, syntenic with that of *M. marinum*, with very few insertion sequences. A vast array of virulence factors includes 283 PE/PPE surface-associated proteins, making up 10% of the coding capacity, and 22 non-ribosomal peptide synthase clusters. A comparison of six clinical isolates from the five patients shows that they differ by up to two single nucleotide polymorphisms, suggesting a common source of infection. Our findings are in accordance with the recognition of a new taxonomic entity. We propose the name *M. basiliense*, as all isolates were found in patients from the Basel area of Switzerland.

## Introduction

The incidence of nontuberculous mycobacteria (NTM) infection has increased over the past decades, with pulmonary disease being the most common clinical manifestation, affecting mainly immunocompromised patients (Donohue and Wymer, 2016; Stout et al., 2016; Sood and Parrish, 2017). This is partly due increased awareness and detection, which has also led to the identification and description of more species within the NTM, with over 180 now listed (Sood and Parrish, 2017; Oren and Garrity, 2018). Many have been phylogenetically characterized using genome sequences (Fedrizzi et al., 2017), with additional genus subdivisions now proposed (Gupta et al., 2018). With the exception of *Mycobacterium tuberculosis* that co-evolved with primates, most mycobacteria are saprophytic bacteria that are evolutionarily adapted to soil or aquatic environments, acting as facultative pathogens in humans.

*Mycobacterium ulcerans* and *M. marinum* are well characterized, slow growing NTM. Strains of *M. ulcerans* are the causative agents of chronic non-healing skin and soft tissue infections, primarily in tropical and sub-tropical countries in Africa and Australia (Guarner, 2018). Clinical isolates of *M. ulcerans* produce variants of a cytotoxic pro-apoptotic toxin (mycolactone), which is considered as the major virulence factor contributing to the non-healing skin lesions (Yip et al., 2007; Tobias et al., 2009). Skin infections caused by *M. marinum* have been described in fish-tank and frog vivarium owners (Johnson and Stout, 2015). Members of *M. marinum* and *M. ulcerans* infect exterior human body sites, as pathogen growth is restricted to a range of 28–33°C (World Health Organization [WHO] et al., 2001).

Genomic studies on mycobacteria have produced reference genomes for key strains of *M. tuberculosis, M. marinum*, and *M. ulcerans* (Cole et al., 1998; Stinear et al., 2007, 2008). These have resulted in the observation that *M. marinum* represents a more ancestral genomic state, as a generalist bacterium able to survive in various environments as well as causing disease, in comparison to *M. tuberculosis*, which has undergone significant genome reduction and reorganization through insertion sequence (IS) expansion to become a specialized pathogen. *M. ulcerans* has undergone a similar process in parallel, and has also acquired a virulence plasmid carrying genes which encode the immunosuppressive polyketide toxin mycolactone (Doig et al., 2012).

Here, we present a novel species of *Mycobacterium* that is related to *M. marinum* and *M. ulcerans*, and has been associated with five cases of NTM pulmonary infection. We describe the clinical and microbiological features of these cases and provide phenotypic and genomic characterization of the infecting mycobacterial isolates. We propose the name *Mycobacterium basiliense* sp. nov., as all isolates were recovered from patients in the Basel area of Switzerland.

## Materials and Methods

### Patient Consent and Bacterial Isolates

Written informed consent was provided by all patients. Mycobacterial cultures were grown from respiratory samples of all five patients in mycobacterial growth indicator tubes (MGIT 960, Becton Dickinson, Heidelberg, Germany). Positive cultures were further analyzed using a Ziehl-Neelsen and auramine-rhodamine staining. After detection of acid-fast rods in microscopy, we used the GenoType Mycobacterium CM (Hain Lifescience, Nehren, Germany) for further species determination. Cell morphology was analyzed after auramine-rhodamine staining. All isolation work was carried out in an ISO 17025 accredited clinical microbiology laboratory using internal controls for growth (ATCC^®^ 27294) and microscopy (Axonlab, Luzern, Switzerland) as standard. Laboratory contamination is highly unlikely, especially as isolates were found sporadically over four years, and also in one case within samples from the same patient.

### Genomic DNA Extraction and 16S rRNA Gene Sequencing

Genomic DNA was extracted by Qiagen EZ1 (Qiagen, Hilden, Germany) using the DNA tissue kit (Qiagen) after inactivation at 95°C for 1 h and disruption in a TissueLyser (Qiagen) for 2 min at highest frequency. For species identification a 1026 bp fragment of the *rrs* gene encoding the 16S rRNA was generated by PCR, using the oligonucleotides Mbak-f283 5′-GAG TTT GAT CCT GGC TCA GGA-3′ and Mbak-r264 5′-TGC ACA CAG GCC ACA AGG GA-3′ adapted from (Boddinghaus et al., 1990). Amplicons were sequenced using Mbak14 (5′- GRG RTA CTC GAG TGG CGA AC-3′), producing reads of up to 750 bp.

### Phenotypic Properties and Matrix-Assisted Laser Desorption Ionization-Time of Flight (MALDI-TOF) Mass Spectrometry (MS)

Growth of type strain 901379 (the first isolate, from 2013) was assessed using 7H10 agar plates + 10% OADC growth supplement, after incubation for 14 days at either 30°C or 37°C. A standard series of biochemical tests and morphological examination were performed as described in (Pfyffer, 2015). MALDI-TOF MS profiles were acquired on a Bruker microflex system and interpreted using the Bruker mycobacterial database (version 3.0) (Detailed methods are in Supplementary File 1).

### Antimicrobial Susceptibility Testing (AST)

Antimicrobial susceptibility testing (AST) was performed using MGIT 960/TB eXiST DST (Becton Dickinson) as previously described for slow growing NTM (Lucke et al., 2012). Briefly, isolates were examined using a modified automated macrodilution assay with defined drug concentrations below and above the epidemiologic cutoff values (ECOFF) of the individual antimicrobial compounds. Tested compounds and the respective concentrations were as follows: clarithromycin at 4, 16, 32, and 64 mg/L; ethambutol at 5, 12.5, and 50 mg/L; rifabutin at 0.1, 0.4, and 2 mg/L; rifampicin at 1, 4, and 20 mg/L; clofazimine at 1 and 2 mg/L; amikacin at 1, 4, and 20 mg/L; moxifloxacin at 0.5, 2.5, and 10 mg/L; linezolid at 1, 4, and 16 mg/L. Growth was monitored with the EpiCenter software and the TB eXiST module (Becton Dickinson). Resistance categories of the isolates (resistant/intermediate/susceptible) were determined as defined (Springer et al., 2009).

### Mycolic Acid Analysis Using High-Performance Liquid Chromatography (HPLC)

Cell-wall mycolic acids were analyzed by HPLC as described recently (Nogueira et al., 2015; Hashemi Shahraki et al., 2017), following the recommendations of the Sherlock Mycobacteria Identification System (SMIS; MIDI). In brief, cells were grown on Middlebrook 7H10 agar, saponified, extracted, and derivatised. Mycolic acids were separated with a gradient of methanol and 2-propanol on an Agilent ChemStation 1100/1200 HPLC system. Peak integration and identification were performed with the MIDI Sherlock Software version 4.0.

### *In vitro* Cell and Amoebal Growth

Detailed methods are given in Supplementary File 1. *M. basiliense* strain 901379 was used to infect both THP-1 human macrophages and *Acanthamoeba castellanii* ATCC 30010 at a multiplicity of infection (MOI) of 10 and 5, by centrifugation at 1790 *g* for 10 min and subsequent incubation for 30 min. Infected THP-1 cells were grown in RPMI supplemented with 10% Fetal Calf Serum at 37°C in a 5% CO_2_ humidified atmosphere. *A. castellanii* were grown at 25°C in Page’s *Amoeba* Saline Solution (PAS) (Jacquier et al., 2013). Samples were taken at 0, 24, 48, 72, and 96 h, after 2 h’ treatment with 150 μg/ml gentamycin, using TrypLE Express (Thermo Fisher Scientific, United States) to detach THP-1 cells.

Amoebal cell suspension or THP-1 cells were diluted in phosphate buffered saline (PBS) and shaken with 4 mm beads to lyse the host cells. Colony-forming units (CFU) from serial dilutions were measured after growth on 7H10 Middlebrook agar plates at 37°C, 5% CO_2_ humidified atmosphere.

For Ziehl-Neelsen staining, 300 μl of cell suspension were spun onto coverslips at 1000 *g* for 5 min. After drying, the coverslips were heat fixated at 95°C for 10 min before staining. In parallel, DNA from infected cells was extracted using RTP^®^ MYCOBACTERIA KIT (STRATEC, Germany; manufacturer’s protocol 2 without the *N*-Acetyl-Cysteine step). Mycobacterial DNA was quantified in duplicate by qPCR method C (Radomski et al., 2010) using a StepOne Real-Time PCR System (Applied Biosystems, Thermo Fisher Scientific, United States).

### Genome Sequencing, Assembly, Annotation and Mapping

All isolates were sequenced following Nextera library creation, on the Illumina Miseq platform using 300 bp paired end reads, within the Division of Clinical Microbiology, University Hospital Basel. DNA extracted from the type strain 901379 was sequenced on the Pacific Biosciences RSII platform using one SMRT cell, at the Functional Genomics Centre Zürich (FGCZ), ETH Zürich. PacBio reads (mean 140-fold coverage) were assembled using the RS_HGAP_Assembly.3 protocol implemented in SMRT Portal version 2.3.0. Illumina reads were mapped onto the resulting contig by using BWA to improve the sequence quality. Annotation was performed using Prokka 1.8 with further manual curation in Artemis (Rutherford et al., 2000) and Artemis Comparison Tool (ACT) (Carver et al., 2005), and the chromosome split at *dnaA* representing *oriC*. The draft genome was curated for the presence of pseudogenes, defined as putative coding sequences (CDSs) disrupted by one or more mutations that would ablate expression (i.e., indel causing frameshift or substitution causing premature stop codon). Mapping of MiSeq data was performed within CLC Genomics Workbench 9, giving mean MiSeq read coverage values of 901379: 52×, 900310: 37×, 903442: 119×, 902333: 61×, and 902167: 47×. This software was also used to perform variant calling using the parameters: 8× min coverage, 8 min count and 50% min frequency, with manual checking of all called variants (The same results were achieved using 10× min coverage, 10 min count and 70% min frequency). A single nucleotide polymorphism (SNP) phylogeny was generated from high quality confirmed SNPs.

### Genome Analysis

Average nucleotide identity (ANI) determination was performed at enve-omics.ce.gatech.edu/ani/ (Goris et al., 2007) and digital DNA-DNA hybridization (dDDH) using GGDC2.1^1^ and the DDH cut off of ≤70% (Auch et al., 2010). For the core-genome phylogeny, translated CDSs were clustered into orthologous groups using OrthoFinder v2.2.1 (Emms and Kelly, 2015) and single-copy orthologous genes were aligned using Mafft v7.310 (Katoh and Standley, 2013). The length of the concatenated alignment of 793 CDSs was 283,167 amino acids. A maximum likelihood tree was built using FastTree v2.1.10 Double precision using parameters *(–gamma –spr 4 –mlacc 2*) (Price et al., 2010), with the tree rooted on *M. abscessus* ATCC 19977 using Archaeopteryx 0.9921^2^ (Zmasek, 2015). Searches were performed using http://www.pathogenomics.sfu.ca/islandviewer/, http://phast.wishartlab.com/, https://cge.cbs.dtu.dk/services/PathogenFinder/ (Cosentino et al., 2013) and the virulence factor database (VFDB) (Chen et al., 2016). Prediction of gene clusters involved in antibiotic and secondary metabolite production used antiSMASH v4.0.0^3^ (Weber et al., 2015).

### Accession Numbers

Read data generated for this study, and the annotated assembly of the type strain 901379, can be found in the European Nucleotide Archive (ENA^4^) under project number PRJEB23647, accession number LR130759.

## Results

### Clinical Cases of New Mycobacterial Species

An undescribed *Mycobacterium* species was first cultured from bronchoalveolar lavage (BAL) samples from patient 1 in 2013. The same *Mycobacterium* sp. was cultured and isolated from BAL and bronchial secretion specimens from four further patients, attending three healthcare providers, in 2016 and 2017. Four of the five patients had underlying lung conditions. A summary of the five case histories is given in Table 1, with detailed case reports in Supplementary File 2. Case reports were made as thoroughly as possible, yet no clear common source of infection (geographic or healthcare provider) could be identified. In four of the cases, infection was associated with cough, hemoptysis and/or dyspnea. Yet only one case fulfilled ATS (American Thoracic Society) guideline criteria, indicative of non-mycobacterial pulmonary disease (Griffith et al., 2007). Treatment was not administered in most cases, yet subsequent samples were culture negative in three cases (of four in which further samples were tested). All six isolates of *Mycobacterium* were diagnosed by the molecular genotyping test (Hain), however, test band patterns indicated an inconclusive result (Genotype 1.2.3.9.10.13.15, the latter variably present) (Lee et al., 2009).

**Table 1:** Clinical characteristics of five patient cases.

Patient (year of birth)	1 (1930)	2 (1951)	3 (1942)	4 (1956)	5 (1939)
Date of first detection	June 2013	January 2016	May 2016	Nov-16	July 2016
Healthcare provider	A	B	A	A	C
Sampling material	BAL	Bronchial secretion	BAL	BAL	Bronchial secretion
Isolate identifier	901379	900310	Unavailable	903442	902167
Symptoms	None	Cough, dyspnea (DD: COPD exacerbation, lung cancer)	Cough, hemoptysis (DD: concomitant bacterial infection with *Staphylococcus aureus*)	Cough (DD: viral LRTI)	Hemoptysis, dyspnea (DD: concomitant bacterial infection with *Pseudomonas aeruginosa*)
Underlying lung disease	None	COPD GOLD III	Pulmonary sarcoidosis	Status after lung transplantation (2007) caused by end stage pulmonary sarcoidosis	COPD
		Squamous cell cancer in RUL (cT4, cN2, cM0; stadium IIIA) treated with radio-chemotherapy	Tuberculosis in childhood (no treatment)		Acute pulmonary embolism
Radiology	Nodular consolidation RUL	Progressive nodular lesion apical RUL and diffuse infiltrations bilaterally	Cavernous consolidations predominant RUL > LUL	Unremarkable/no specific lesions	Ground glass opacities, infiltration
Cytology/histology	Histology: Multiple caseating epithelioid granulomas	Cytology (brush): Dysplastic squamous cells	Histology: Non-necrotising granulomatous inflammation	Cytology (BAL): moderate granulocytosis	Cytology (BAL): granulocytosis
Additional cultures	September 2013 (lung biopsy): negative culture, Genus-specific PCR positive	February 2016 (BAL/secretion): negative; September 2016 (secretion): positive, 902772; January 2017 (sputum): negative	August 2016 (sputum): positive, 902333	January 2017 (BAL): negative February 2017 (BAL): negative	None
ATS criteria	**Clinical:** yes (CT scan with nodular opacity without other diagnoses) **Microbiological:** yes (positive culture from lavage AND biopsy with mycobacterial histopathologic features)	**Clinical:** uncertain (nodular opacity in the ct scan and symptoms probably caused by lung cancer and COPD, respectively) **Microbiological:** yes (positive cultures from lavage)	**Clinical:** uncertain (cavitary opacity in the CT scan probably caused by pulmonary sarcoidosis; cough and hemoptysis probably caused by proven bacterial infection) **Microbiological:** yes (positive cultures from lavage and sputum AND biopsy with mycobacterial histopathologic features)	**Clinical:** uncertain (pulmonary symptoms probably caused by viral respiratory tract infection) **Microbiological:** yes (positive culture from lavage)	**Clinical:** uncertain (pulmonary symptoms probably caused by pulmonary embolism and proven bacterial infection) **Microbiological:** yes (positive culture from lavage)
Treatment	Resection; no antibiotics	None	Amikacin, clarithromycin, rifampicin, and ethambutol was established but stopped after 6 weeks because of multiple side effects	None	Patient unfortunately died due to respiratory failure as result of the acute thromboembolic event
Possible epidemiological links	Several holiday trips, mainly to Italy, but not in 10 years prior to diagnosis	Several holiday trips to south of Italy	Lives partly in Sicily, Italy	Worldwide travel	Traveled, mainly to the south of Italy

*COPD, chronic obstructive pulmonary disease; DD, differential diagnosis; GOLD, Global Initiative for Chronic Obstructive Lung Disease; RUL, right upper lobe; BAL, bronchoalveolar lavage; LUL, left upper lobe; PCR, polymerase chain reaction; LRTI, lower respiratory tract infection; ATS, American Thoracic Society guidelines for Nontuberculous Mycobacterial Diseases (Griffith et al., 2007). Healthcare providers A, B and C are all within North–West Switzerland.*

### Identification by 16S rRNA Gene Sequencing

Species identification was attempted by sequencing a 16S rRNA gene fragment covering the hypervariable regions A and B, which are used for mycobacterial species identification (Boddinghaus et al., 1990). The six isolates possess identical 16S rRNA gene sequences, with top Blastn hits (nr/nt and 16S rRNA databases) to *M. marinum* (AB716939; 767/771, 99.5%) and *M. ulcerans* (AB548729; 767/771, 99.5%), as these latter species have identical sequences over this region.

### Phenotypic Characterization of Novel Strain

**FIGURE 1:**
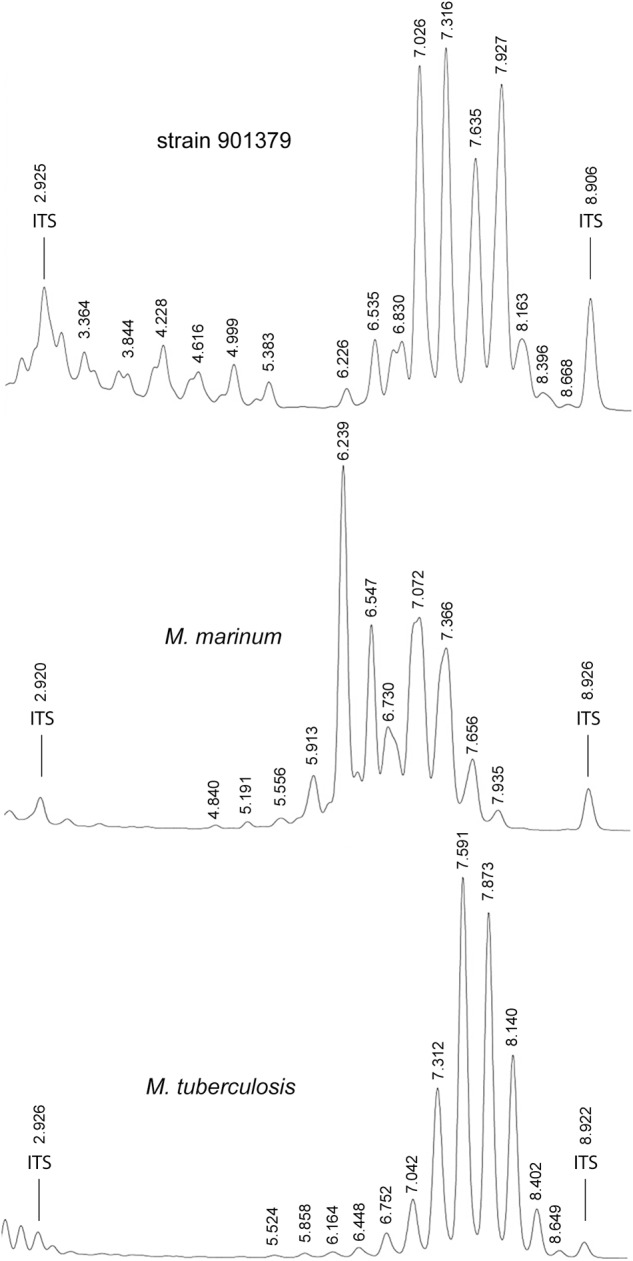
Representative high-performance liquid chromatography (HPLC) patterns showing cell-wall mycolic acid content of *Mycobacterium basiliense* in comparison with *M. marinum* and *M. tuberculosis*. Numbers indicate retention times (minutes). ITS, internal standard.

Bacteria were cultivated from six respiratory samples from five patients. Ziehl-Neelsen and auramine-rhodamine staining showed acid-fast, 3.2 ± 0.6 μm long, rod-shaped bacilli. Cords, spores or filaments were not observed. Growth of strain 901379 on Middlebrook 7H10 agar plates at 30°C showed non-photochromogenic, white, slightly undulated colonies, apparent after approximately 10 days of incubation, making this a slow growing *Mycobacterium* (Brown-Elliott and Wallace, 2002). The biochemical characteristics of strain 901379 were similar to those of *M. marinum* and *M. ulcerans* (Table 2). The most striking exception is its ability to grow at 37°C. AST revealed full susceptibility of this strain to all antibiotics tested (Table 3). Furthermore, phenotypic detection of pyrazinamidase (Table 2) indicates that the strain is sensitive to pyrazinamide. Analysis of the cell wall mycolic acids by HPLC demonstrated a unique profile for strain 901379, clearly distinct from those of *M. marinum* and *M. tuberculosis* (Figure 1).

**Table 2:** Biochemical and growth characteristics of *M. basiliense*.

Characteristics	*M.basiliense*	*M. marinum^∗^*	*M. ulcerans^∗^*	*M. tuberculosis^∗^*
Growth at 30°C	+	+	+	+
Growth at 37°C	+	–	–	+
Photochromogenicity	N	P	N	N
Nitrate reduction	–	–	–	+
Pyrazinamidase^∗∗^	+	+	–	+
Urease	+	+	V	±
Tellurite reduction	+	–/+	–	–/+
TWEEN 80 hydrolysis	–	–	–	±
Growth on MacConkey agar	–	–	–	–

*+, positive; -, negative; ±, usually present; -/+, usually absent; V, variable; P, photochromogenic; N, non-chromogenic; ND, not determined. ^∗^Data for M. marinum, M. ulcerans, and M. tuberculosis were retrieved from literature (Murray et al., 2007). ^∗∗^Identified in the genome as MB901379_02799.*

**Table 3:** Antimicrobial susceptibility testing.

Antibiotic	Concentration range tested (mg/l)	MIC (mg/l) for analyzed isolates	Interpretation
Clarithromycin	4–64	≤4–16	S
Rifampicin	1–20	≤1–4	S
Rifabutin	0.1–2	≤0.1–0.4	S
Ethambutol	5–50	<5	S
Amikacin	1–20	≤1–4	S
Moxifloxacin	0.5–10	≤0.5	S
Clofazimine	1–2	≤1	S
Linezolid	1–16	≤1–4	S

*Isolates were tested in MGIT 960 supplemented with the indicated antibiotics. Strain 901379 was susceptible to the lowest concentration tested of all antibiotics.*

MALDI-TOF MS routine identification did not produce a clear result (“No Identification Possible”). A consensus mass peak profile of all *M. basiliense* isolates was generated and compared to peak profiles of *M. marinum*; no *M. basiliense*-specific peaks were identified. A representative mass spectrum of *M. basiliense* is given in Supplementary Figure S1.

### Intracellular Growth in Macrophages and Amoebae

**FIGURE 2:**
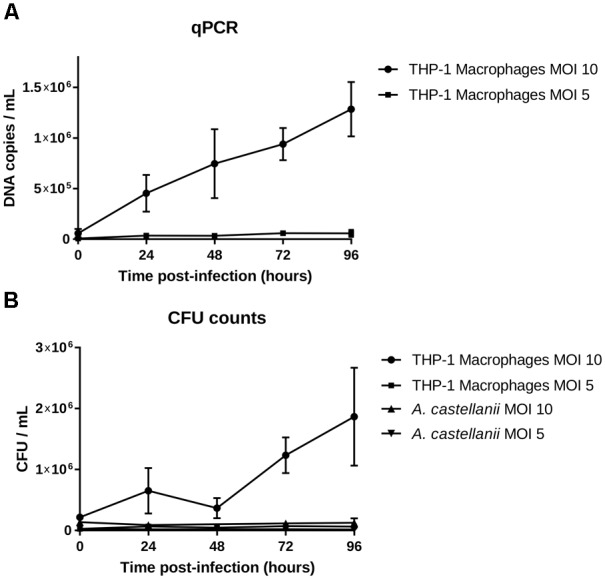
Growth in THP-1 human macrophages and in *Acanthamoeba castellanii* ATCC 30010. **(A)** DNA copies/ml over the growth time (means ± 2 standard deviations). **(B)** CFU/ml over the growth time (means ± 2 standard deviations).

**FIGURE 3:**
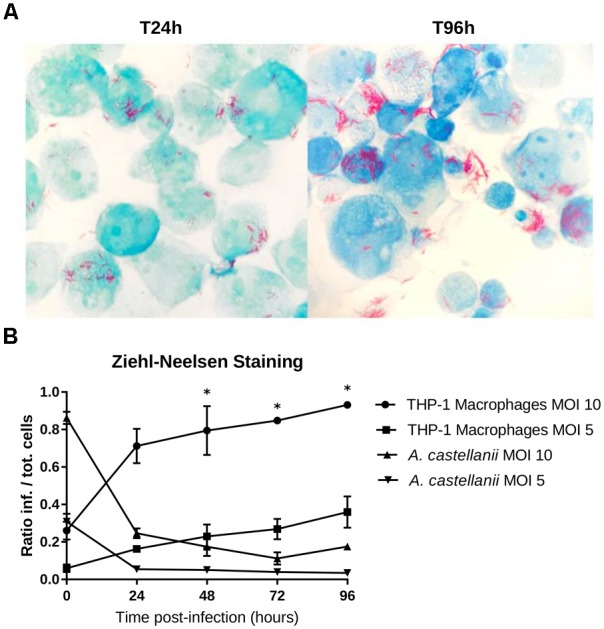
THP-1 macrophage infection shown using Ziehl-Neelsen staining. **(A)** THP-1 cells strained with ZN 24 and 96 h post-infection with an MOI of 10. Mycobacteria were observed in the extracellular medium, probably explained by recent host cell lysis events; extracellular growth is unlikely to have occurred because infected cells were treated with gentamycin to remove bacteria that were not internalized. **(B)** Ratio of the infected cells to the total number of cells counted (means ± 2 standard deviations). The asterisks (^∗^) indicate the timepoints at which each condition was significantly different from another by doing pair-wise chi-squared tests between pairs of triplicates with five degrees of freedom (*p*-value < 0.05).

Isolate 901379 was able to grow in phagocytic THP-1 macrophages when infected at an MOI of 10, as documented by PCR and by counting CFUs (Figures 2A,B). Ziehl–Neelsen staining showed an increased ratio of infected THP-1 cells over the time course of the infection (Figure 3A). However, at MOI 5 in THP-1 cells, growth was limited.

No growth was detected in the amoebae *A. castellanii* when infected at MOI 10 or 5 by qPCR, CFU counting or microscopy. Ziehl–Neelsen staining also showed a reduced percentage of infected cells over time (Figure 3B), indicating that mycobacteria did not survive internalization.

### A Novel Species of *Mycobacterium* Defined by Genomics

**FIGURE 4:**
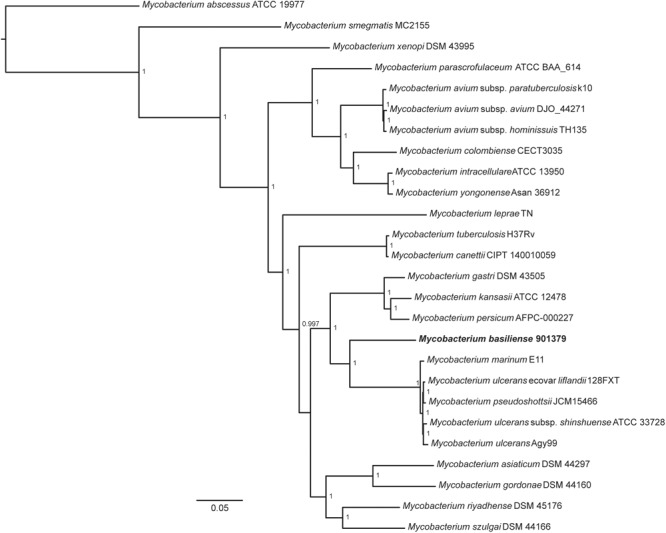
Core genome phylogeny of slow growing mycobacterial species. *M. basiliense* can be seen to be most closely related to *M. ulcerans, M. marinum, M. ulcerans* ecovar *liflandii* and *M. pseudoshottsii*, as well as to the *M. kansasii* complex. Shared core genes (*n* = 768) were obtained through clustering CDSs into orthologous groups using OrthoFinder v2.2.1 (Emms and Kelly, 2015). Single-copy orthologous genes were aligned using Mafft v7.310 (Katoh and Standley, 2013), resulting in a concatenated alignment of 283,167 amino acids. A maximum likelihood tree was built using FastTree v2.1.10 Double precision using parameters *(–gamma –spr 4 –mlacc 2*) (Price et al., 2010). The tree was rooted on *M. abscessus* ATCC 19977 using Archaeopteryx 0.9921 (Zmasek, 2015) (https://sites.google.com/site/cmzmasek/home/software/archaeopteryx). The scale bar represents the number of amino acids substitutions per site alongside the branches. Nodes supports are based on the Shimodaira-Hasegawa (SH) test. NB, BLAST (using BLASTn) of the *M. shottsii*-specific F5 fragment (accession number HM149249) against the *M. basiliense* assembly returned no hit.

A single scaffold of 5.6 Mb was obtained through hybrid assembly of PacBio and Illumina sequencing data, representing the chromosome of strain 901379. A phylogenetic tree comparing the core genome of strain 901379 to that of other slow growing mycobacteria (Figure 4) shows that it is divergent from other species, and positioned most closely to *M. ulcerans* and *M. marinum.* Comparison of the genome assembly with those from closely related mycobacterial species was performed using average nucleotide identity (ANI) and digital DNA:DNA hybridization (dDDH) (Table 4), with both methods indicating that strain 901379 represents a new species of *Mycobacterium*. We have named this species *M. basiliense* sp. nov., as all the patients were diagnosed in the Basel area of Switzerland.

**Table 4:** Comparison of the genome of strain 901379 other mycobacterial genomes.

Reference genome, Accession	ANI (%), two way	dDDH	Prob. DDH ≥ 70%	%G+C difference
*M. ulcerans* ecovar *liflandii* 128FXT CP003899	81.15	23.3	0	0.59
*M. marinum* M CP000854	81.14	23.2	0	0.7
*M. pseudoshottsii* JCM 15466 AP018410	81.09	23.2	0	0.62
*M. ulcerans* Agy99 CP000325	81.00	23.2	0	0.45
*M. tuberculosis* H37Rv CP009480	79.99	22.3	0	0.58

*New species are described with an ANI < 95% and dDDH < 70%. ANI was calculated at http://enve-omics.ce.gatech.edu/ani/ (Goris et al., 2007) and dDDH at http://ggdc.dsmz.de/ggdc.php (Auch et al., 2010) using the results of formula 2.*

### Genome Overview

**FIGURE 5:**
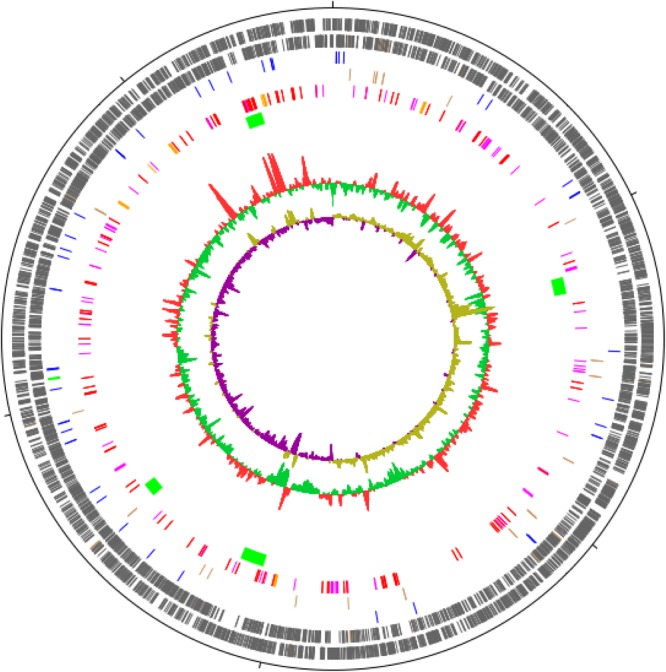
Circular map of the draft genome of *M. basiliense* strain 901379. The 5.6 Mb chromosome is represented as a circle, with ticks every 1 Mb. The circles represent, from outside to inside: CDSs encoded on the forward strand; CDSs encoded on the reverse strand; tRNAs (blue) and rRNAs (green); pseudogenes (brown); CDSs encoding PE (red), PPE (pink) and Mce (orange) proteins; regions not present in *M. marinum* strain M (green); G+C content; G+C skew. Figure was produced in DNAPlotter (Carver et al., 2009).

The annotated genome draft of *M. basiliense* comprises 5,607,630 bp, containing 4,817 predicted coding sequences (CDSs) (Figure 5 and Table 5). This is on a par with the size of the *M. ulcerans* genome (Stinear et al., 2007), 1 Mb smaller than that of the “more ancestral” *M. marinum* (Stinear et al., 2008) and 1.2 Mb larger than that of *M. tuberculosis* (Cole et al., 1998). No evidence of a plasmid was found, with over 99% of MiSeq reads mapping to the assembled chromosome. Through curation of PHAST and Island Viewer results, the presence of four phages was predicted, carrying cargoes of genes encoding hypothetical proteins and a restriction modification system (Supplementary Table S1), indicating that *M. basiliense* has the capacity for lateral gene transfer. The single ribosomal RNA operon is located 1.5 Mb distant from the origin of replication, which is thought to be associated with slow growth in *M. tuberculosis* (Cole et al., 1998).

**Table 5:** Genome features of *M. basiliense* strain 901379.

Draft genome size	5607630 bp
% G+C content	65
Predicted CDSs	4817
Coding density	0.86
Average gene length	1048
Pseudogenes	19
rRNA operons	1
tRNAs	53

**FIGURE 6:**
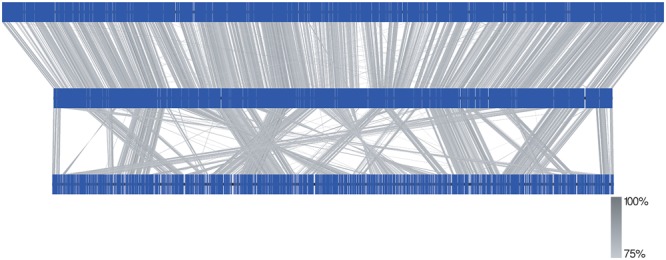
Comparison of the genomes of *M. basiliense* 901379 **(middle)** with *M. marinum* M (CP000854, **top**) and *M. ulcerans* Agy99 (CP000325, **bottom**). Genomes are depicted linearly with predicted CDSs as blue vertical lines. Blastn hits between the genomes (identity shown in scale) show that the genome of *M. basiliense* is syntenic with that of *M. marinum*, whereas the genome of *M. ulcerans* contains many rearrangements. Figure was drawn using Easyfig (Sullivan et al., 2011).

A comparison against the genomes of *M. marinum* and *M. ulcerans* shows that the genome of *M. basiliense* has retained synteny with *M. marinum* (Figure 6). Four large insertions are apparent, three of which encode for T1pks systems (see below), and the fourth contains an array of genes encoding PE/PPE proteins, named after conserved N terminal proline-glutamate (PE) or proline-proline-glutamate (PPE) motifs. Regions of the *M. marinum* genome which are absent from *M. basiliense* comprise up to 1Mb of sequence and include: three polyketide synthase clusters; five non-ribosomal peptide synthase clusters; seven putative phages; several transposases; several genes encoding virulence proteins within the families PE, PPE and mce (mammalian cell entry); and various dehydrogenase, mono-oxygenase and P450 encoding genes, which may be involved in metabolite hydroxylation or oxidation of xenobiotics (Stinear et al., 2008).

Through manual curation of the *M. basiliense* genome annotation, in comparison to that of *M. marinum* strain M, 19 putative pseudogenes were identified (Supplementary Table S2). This is fewer than the 65 annotated in *M. marinum* (Stinear et al., 2008), whereas *M. ulcerans* possesses 771 (Stinear et al., 2007). In a similar vein, *M. basiliense* possesses only seven CDSs annotated as transposases, representing possibly five insertion sequences (IS), and with no signs of IS expansion, whereas the genomes of *M. ulcerans* and *M. tuberculosis* contain expanded IS families, which can also have a gene-disruptive effect. This all indicates that *M. basiliense*, along with *M. marinum*, represents a more ancestral genomic organization and content than *M. ulcerans* and *M. tuberculosis*.

### Pathogenic Potential and Virulence

Genes encoding common virulence associated proteins within the mycobacteria are also found within the genomes of strain 901379. The vast majority of mycobacterial virulence factors, as defined within the virulence factor database (Chen et al., 2016), were identified within the genome of strain 901379 (Supplementary Table S3), and *M. basiliense* was predicted to be a human pathogen by PathogenFinder, with a probability of 0.89 (Cosentino et al., 2013).

The PE/PPE family comprise specific mycobacterial virulence factors, thought to be involved with antigenic variation and immunopathogenicity (Cole et al., 1998; Stinear et al., 2008). The genome of strain 901379 contains 202 and 81 CDS members of PE/PPE families, respectively, representing 10% of the coding potential of the genome, on a par with the genome of *M. marinum*, and possibly indicative of a diverse host range. These proteins are exported through the ESX/Type VII secretion system (Simeone et al., 2015), of which components are found in several gene clusters throughout the genome. Another family of virulence proteins, well represented within the genome of strain 901379 is that of *mce*, associated with the invasion process and prolonged existence in host macrophages (Zhang and Xie, 2011). There are 30 genes encoding members of this family, organized within five clusters, similar to the 29 found within *M. marinum*. In addition, three haemolysins have been predicted: *MB901379_02511, 02512, 03838*, along with many other genes involved in antigen and immunogenic protein production.

Polyketide synthesis operons and others involved in non-ribosomal peptide synthesis (NRPS) are known to be common in the genomes of mycobacteria, responsible for the production of cell wall-associated lipids, siderophores and other biologically active molecules (Cole et al., 1998; Stinear et al., 2008). A list of 22 such gene clusters identified within *M. basiliense* is given in Supplementary Table S4. The presence of over 200 predicted regulators shows the versatility of *M. basiliense* in gene expression, with 19 sigma factors and over 30 serine/threonine protein kinases, comparable with *M. marinum*.

Of genes identified as being putatively involved in drug resistance, several did not have homologs in *M. marinum*, in particular the MFS transporters (*MB901379_00088, 01067, 02557, 03180, 03953*), and the ABC transporter encoding operon *MB901379_00248-50*. Although *M. basiliense* has low minimum inhibitory concentrations (MIC) to all tested antibiotics, there are CDSs predicted to encode six putative aminoglycoside phosphotransferase, one aminoglycoside acetyltransferase, 11 putative drug efflux pumps, eight putative beta-lactamases, and over 30 additional CDSs annotated as being involved in ABC transport.

### Variation Between Clinical Isolates of *M. basiliense*

**FIGURE 7:**
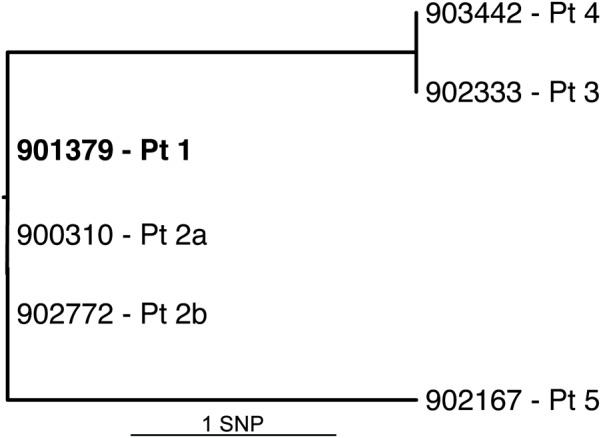
Genome phylogeny of six *M. basiliense* isolates. SNP phylogeny of the full genome based on high quality, manually checked SNPs. Root is assigned to the type strain (earliest isolate, bold), which was also the mapping reference. Additional isolates, identified by isolate number and patient, vary by a maximum of two SNPs from the root.

All six isolates of *M. basiliense*, from five patients, were subjected to whole genome sequencing. Variants were identified between these genomes using variant calling followed by careful manual checking. The isolates were found to vary by a maximum of two SNPs from the type strain (Figure 7), after excluding SNPs within repetitive NRPS genes, which were attributed to imperfect mapping. These SNPs are located in *MB901379_01152* (synonymous) and the regulator *MB901379_01152*, causing A174G, in isolates 902333-16 and 903442-16. Isolate 902167-17 has two non-synonymous SNPs, causing V555G in putative ribonuclease MB901379_01783, and elimination of the STOP codon of the PPE family member MB901379_02461, extending the C terminus by 62 residues. This isolate also possesses a 3 bp duplication at the 3′ end of *MB901379_03561*, encoding ATP synthase I chain. Other than this, no further genomic differences between the six isolates could reliably be called. The two isolates from the same patient, separated by a 9-month period between sampling, are identical.

Few estimates exist for mutation rates of mycobacterial species, with that of *M. tuberculosis* estimated at 0.3–0.5 mutations per genome per year over a 10-year period (Ford et al., 2011, 2013), and no temporal signal identified in a study of *M. ulcerans* (Doig et al., 2012). The *M. basiliense* isolates show 0–2 SNPs occurring over 3–4 years, which would fit with the above estimate, assuming a possibly common source of infection, although there is no epidemiological evidence for this. This low rate may be associated with a slow replication rate and an unknown environmental reservoir.

### Taxonomic Description of *Mycobacterium basiliense* sp. nov

A slow growing, acid-fast, rod-shaped bacillus (901379) was isolated from human bronchoalveolar lavage. On the basis of 16S rRNA gene sequence similarity, strain 901379 was shown to belong to mycobacterial genus of actinobacteria, most closely related to both *M. marinum* (99.7%) and *M. ulcerans* (99.5%). Physiological and biochemical characterization revealed the following traits for the isolate: growth at 37°C, no photochromogenicity, nitrate reduction negative, pyrazinamide positive, urease positive, tellurite reduction positive, TWEEN 80 hydrolysis negative, no growth on MacConkey agar (Table 1). HPLC analysis of cell-wall mycolic acids revealed a pattern clearly distinct from its close relative *M. marinum*. The results of digital DNA–DNA hybridization and ANI allowed genotypic differentiation of strain 901379 from the validly published mycobacterial species. Strain 901379 therefore represents a new species, for which the name *M. basiliense* sp. nov. (ba.si.li’en.se. N.L. neut. adj. basiliensis pertaining to the city of Basel, Switzerland) is proposed, with the type strain 901379 (=DSM 104308 = CCOS1136 = CIP 111488T).

## Discussion

We describe a new species of slow growing *Mycobacterium*, which we have named *M. basiliense*. The genome of *M. basiliense* is rich in virulence factors, as well as being highly intact, resembling in structure more that of *M. marinum*, than *M. ulcerans* or *M. tuberculosis*, and possibly representing a genome of a common ancestor. The latter two species are highly specialized for specific pathogenic niches, illustrated by genomes which have undergone reduction and which, in the case of *M. ulcerans*, is replete with IS elements and pseudogenes, and has gained a virulence plasmid. From this we can predict that *M. basiliense* is a generalist, capable of environmental/extracellular survival, but is potentially pathogenic with likely intracellular persistence and a possibly diverse host range.

Although the genomic findings are suggestive of a pathogen, the clinical description indicates that the infection is not severe, and may just represent colonization. *M. basiliense* was isolated from human respiratory samples, yet the clinical significance is unclear as the bacterium appeared to clear in several cases without treatment. Associated symptoms may include cough, even hemoptysis, and shortness of breath, although these were observed in patients with underlying lung disease. Interesting phenotypic features of *M. basiliense* include the ability to grow at 37°C; sensitivity to all tested antibiotics could indicate a lack of previous exposure of these isolates to such drugs.

The ability to grow in human macrophages is indicative of pathogenic behavior, as is the inability to grow in *A. castellanii* (Greub and Raoult, 2004). This latter finding also suggests that the reservoir of these bacteria is not aqueous, in contrast to many other NTM such as *M. avium* (Danelishvili et al., 2007) and *M. kansasii* (Goy et al., 2007), although a wide diversity of amoeba can be encountered in aqueous environments, and we tested only one species.

It is interesting that *M. basiliense* has not previously been identified and characterized, and that all isolates of this species to date were found in the area around Basel, Switzerland. The genotype (Hain) of this species is unique, as is the 16S rRNA gene sequence, and we anticipate that this species will be found more widely with this new data and interpretation. The patients were treated at three healthcare centers, which would appear to rule out the possibility of these isolates having arisen somehow as a contaminant.

The transmission and source of these cases is unknown; that the isolates we have studied are so closely related may indicate a common source of infection for these cases, which we could not identify from case reports. In the case of *M. basiliense*, the genome content suggests a higher virulence potential than is seen in the self-limited infections described, meaning that the potential of this as an emerging pathogen is also unknown. This novel species opens up more possibilities for investigations in mycobacteria, and may represent a useful model for future mycobacterial studies.

## Author Contributions

AE and PK designed the experiments. VB, KJ, AT, PG, SE, MT, GR, and MO diagnosed and described the cases. FI, RH, and PK performed phenotypic characterization. SD and CC performed mycolic acid analysis. AC performed the MALDI-TOF analysis. FT and GG performed the infection experiments. MR, SB, and SG performed high molecular weight DNA extraction. UN performed the assembly and annotation of PacBio data. HS-S performed the genomic analysis. HS-S, FI, PK, CC, FT, GG, SG, and AE wrote the manuscript.

## Conflict of Interest Statement

The authors declare that the research was conducted in the absence of any commercial or financial relationships that could be construed as a potential conflict of interest.

## Footnotes

1 http://ggdc.dsmz.de/ggdc.php#

2 https://sites.google.com/site/cmzmasek/home/software/archaeopteryx

3 https://antismash.secondarymetabolites.org/

4 http://www.ebi.ac.uk/ena/
