# Supplementary material for: Discovery and Characterization of Mycobacterium basiliense sp. nov., a Nontuberculous Mycobacterium Isolated From Human Lungs
Source: Front Microbiol. 2019 Jan 8;9:3184. doi: 10.3389/fmicb.2018.03184 (PMC6331445; doi:10.3389/fmicb.2018.03184)
Supplement: Supplementary file 1 [file Data_Sheet_1.pdf]

Supplementary Material

Discovery and characterization of *Mycobacterium basiliense* sp. nov., a nontuberculous mycobacterium isolated from human lungs

Helena M.B. Seth-Smith[1,2*†], Frank Imkamp[3†], Florian Tagini[4], Aline Cuénod[2], Rico Hömke[3,5], Kathleen Jahn[6], Anne Tschacher[7], Peter Grendelmeier[7], Veronika Bättig[8], Stefan Erb[8], Miriam Reinhard[9,10], Gottfried Rütimann[11], Sonia Borrell[9,10], Sebastien Gagneux[9,10] , Carlo Casanova[12], Sara Droz[12], Michael Osthoff[9,13], Michael Tamm[6], Ulrich Nübel[14,15], Gilbert Greub[4], Peter M. Keller[3,5†], Adrian Egli[1,2*†]

*** Correspondence:** Corresponding Authors: Adrian.egli@usb.ch, helena.seth-smith@usb.ch

Supplementary File 1: supplementary methods

MALDI-TOF MS Sample preparation

For Matrix-Assisted Laser Desorption Ionisation-Time Of Flight (MALDI-TOF) MS measurements, samples were prepared according to Bruker's *Mycobacteria* Extraction protocol (MycoEX) (version 3). *Mycobacteria* Growth Indicator Tubes (MGIT 960, Becton Dickinson, Heidelberg, Germany) were left to rest for 15 min in order to concentrate the bacterial material at the bottom of the tube. 1.25 ml were aspirated from the bottom of the tube and transferred to a 1.5 ml screw tube. The samples were centrifuged for 2 min at 13,000g, the supernatants were removed and the pellets were resuspended in 300 µL ddH$_2$O. The samples were heat inactivated for 60 min at 95°C on a heat block. 900 µL 100% EtOH was added to the screw tubes which were then vigorously vortexed. The samples were centrifuged for 2 min at 13,000g, the supernatants were removed and the samples were centrifuged again for 2 min. The residual supernatants were carefully removed by pipetting and the samples were left to dry completely at room temperature. Subsequently, a small spatula of 0.5 mm-diameter glass beads was added to each tube. 25 µL acetonitril was added to the tubes which were subsequently vortexed for 1 min at maximal speed. 25 µL of 70% formic acid was added and the tubes were vortexed again for 5 seconds at maximal speed. 1 µL of each supernatant was transferred to the MALDI-TOF MS target steel plate and left to dry at room temperature. Once dry, the spots were overlaid with 1 µL of α-cyano-4-hydroxy

cinnamic acid (CHCA) matrix solution and left to dry at room temperature. MALDI-TOF MS profiles were acquired on a Bruker microflex system from all isolates and interpreted using the Bruker mycobacterial database (version 3.0).

Intracellular growth in macrophages and amoebae

THP-1 monocytes

THP-1 monocytes were grown at 37°C in a 5% CO_2 humidified atmosphere in cell culture flasks filled with 10ml Gibco® RPMI 1640 Media (Thermo Fisher Scientific, USA) supplemented with 10% Fetal Calf Serum (FCS, warmed at 56°C for 1h before use). To induce differentiation into M1 macrophages, 100ng/ml of phorbol myristate acetate (PMA) was added to a cell suspension of $2x10^5$ cells/ml. One milliliter per well was distributed in 24-wells plates and incubated overnight. Cells were washed twice with RPMI (+ 10% FCS) and kept for 5 days at 37°C in a 5% CO_2 humidified atmosphere. Finally, media were replaced 2h prior to the infection.

Acanthamoeba castellanii

First steps were done as described by Jacquier et al. (1). Briefly, *A. castellanii* ATCC 30010 were grown at 25 °C in cell culture flasks filled with 30ml Peptone-Yeast Extract medium. Flasks were vigorously shaken to harvest the amoeba. Cell suspensions were centrifuged at 1000 *g* for 10min. The pellets were washed twice with Page's amoeba saline (PAS). 1ml per well of a 10^5 cells/ml suspension was distributed in 24-wells plates and incubated for two hours at 25°C to allow the attachment of amoebae.

Infection procedure

Mycobacterium basiliense strain 901379 was grown in MGIT with BACTEC MGIT supplement (Becton Dickinson, USA) at 37°C until positivity. Then, the culture media were centrifuged at 13,000*g*, and pellets were washed twice with Phosphate Buffer Saline (PBS). The cell suspensions were then passed through a 5 µm filter to remove clots. Dilutions were then performed to reach a McFarland of 1, which corresponds to 2×10^8 CFU/ml according to preliminary experiments. Subsequently, the solution was diluted to reach theoretical MOI of 10 and 5 in PAS or RPMI supplemented with 10% FCS media, for *A. castellanii* and THP-1 macrophages, respectively. Culture media were replaced with 1ml of the suspension, centrifuged at 1790*g* for 10min and incubated for 30min at 25°C for *A. castellanii* and at 37°C

in a 5%-CO_2 humidified atmosphere for THP-1 cells. Cells were washed twice with culture media. Then, each well was filled with 1ml of a solution of 150µg/ml gentamicin and PAS or RPMI + 10% FCS for 2h (depending on the cell type) to keep only internalized mycobacteria. Finally, media were replaced with PAS or RPMI + 10% FCS and the first time-points were taken at that stage. Time-points for 0h, 24h, 48h, 72h and 96h were done in three biological replicates. For experiment in THP-1 cells, 1ml of TrypLE Express (Thermo Fisher Scientific, USA) was added to the cell medium and incubated for 30min to detach the cells. Negative controls, undergoing the same procedure but without the infection, were used for each replicate.

Ziehl-Neelsen staining

Three hundred microliters of cells were placed in a Cytospin™ 4 cytocentrifuge (Thermo Scientific, Thermo Fisher Scientific, USA) and spun at 1000*g* for 5 min. Then, after drying the coverslips under the flow hood, we heat fixated them at 95°C for 10min. Coverslips were covered with Ziehl fuchsin and the dye was heated using a flame until the appearance of vapor. After 5min of incubation at room temperature, the coverslips were rinsed with distilled water, then covered with 3% hydrochloric acid solution in isopropanol for 2min and rinsed again with distilled water. Coverslips were covered with methylene blue for 30sec and rinsed again with distilled water. For each replicate, we counted the infected and non-infected cells in 4 different microscopic fields at 1000x magnification.

Colony forming unit

One hundred microliters of amoebal cell suspension (or 200 µl for THP-1 cells) were diluted in 900µl (or 800µl) of PBS and were shaken with 4 mm beads to lyse the host cells. Serial dilutions were performed and colony-forming units were counted after growth on 7H10 agar plates at 37°C, 5% CO_2 humidified atmosphere.

DNA extraction and quantitative real-time PCR

Wells were scraped off using a 200-µl micropipette and the recovered cells were stored at –20°C until DNA extraction. Positive (plasmid) and negative controls (water) were performed in parallel. The RTP® Mycobacteria kit (STRATEC, Germany) was used to extract DNA. We followed the protocol 2 provided by the manufacturer and skipped the N-Acetyl-Cysteine step. To quantify DNA, we followed method C described by Radomsky et al (2). Each sample was analyzed in duplicates using a StepOne Real-Time PCR System (Applied Biosystems,

Thermo Fisher Scientific, USA).

References

1. Jacquier N, Aeby S, Lienard J, Greub G. Discovery of New Intracellular Pathogens by Amoebal Coculture and Amoebal Enrichment Approaches. JoVE J Vis Exp. 2013 Oct 27;(80):e51055–e51055.
2. Radomski N, Lucas FS, Moilleron R, Cambau E, Haenn S, Moulin L. Development of a Real-Time qPCR Method for Detection and Enumeration of Mycobacterium spp. in Surface Water. Appl Environ Microbiol. 2010 Nov;76(21):7348–51.

Supplementary File 2: case histories

Case history 1 (Patient 1, 1930)

In a previously healthy 83-year-old, nodular lesions of the right upper lobe (RUL) were incidentally detected. A subsequent bronchoscopy sample showed growth of a previously undescribed *Mycobacterium*. For clarification, a wedge-resection of the lung was performed. Histopathology of biopsy material showed caseating epithelioid granulomas and acid-fast rod-like bacteria. Standard and mycobacterial culture from the biopsy material was negative, although *Mycobacterium* genus-specific PCR was positive. Without further treatment, the patient presented in a follow-up 3 years later asymptomatic and in a good condition. The patient was born in and lives in Switzerland with several holiday trips mainly to Italy, but not within the 10 years prior to diagnosis.

Case history 2 (Patient 2, 1951)

A 65-year-old woman with a history of chronic obstructive pulmonary disease (COPD) and lung cancer underwent bronchoscopy because of persistent cough, dyspnea and a progressive nodular lesion in the right upper lobe (RUL). Bronchoalveolar lavage (BAL) culture produced growth of a so far undescribed *Mycobacterium*. Follow-up culture of BAL four weeks later was negative, but infection with this *Mycobacterium* spp. could be verified in a bronchial secretion nine months after first detection. Specific antimycobacterial treatment was never implemented as the clinical relevance was uncertain, and symptoms could be retrospectively explained by progressive lung cancer. The patient was born in and lives in Switzerland with several holiday trips to the south of Italy.

Case history 3 (Patient 3, 1942)

A 68-year-old woman with histological proven sarcoidosis in 1993 (not-immunocompromised) and tuberculosis in her early childhood presented with productive cough and intermittent hemoptysis. In the CT scan diffuse cavitary lesions and consolidations were present with a predominant distribution in the left upper lobe (LUL). Culture of the BAL showed a not yet described *Mycobacterium* species related to *Mycobacterium ulcerans/ marinum*. As a relevant infectious bystander *Staphyloccus aureus* was also detected and treated. Follow-up sputum was positive for the *Mycobacterium* spp. Because of persistent cough, an antimycobacterial antibiotic treatment with amikacin, clarithromycin, rifampicin and ethambutol was established, but was stopped after six weeks because of multiple side effects. At that time the patient was asymptomatic, and pulmonary consolidations had significantly improved. She was born in Switzerland, but lives partly in Sicily, Italy.

Case history 4 (Patient 4, 1956)

A 59-year-old lung transplanted patient presented with symptoms consistent with a viral lower respiratory tract infection (LRTI). The CT scan was significant only for non-specific pulmonary lesions. The patient underwent BAL to differerentiate between infection and transplant rejection. Standard culture and PCR testing for respiratory viruses was negative, but mycobacterial cultures were again positive for a not yet described *Mycobacterium* species. The follow up BAL cultures two and three months later were negative. Because of the uncertain significance of the positive culture and the immediate full recovery treatment was withheld. The patient lives in Switzerland and reports having travelled a lot around the world during his whole life.

Case history 5 (Patient 5 , 1939)

A 88-year-old patient with COPD was admitted with shortness of breath. His past medical history was significant for polyarthritis, which was treated with prednisone and methotrexate. Results of a CT scan were consistent with a diagnosis of acute pulmonary embolism. Additionally, diffuse ground glass opacities were present in both lungs. Bronchoscopy was performed and BAL standard and mycobacterial cultures were positive for *Pseudomonas aeruginosa* and acid fast bacilli with no further differentiation, later identified as a new *Mycobacterium* species. Unfortunately the patient died due to respiratory failure as result of the acute thromboembolic event. The patient was born in Switzerland and travelled also mainly to the south of Italy.

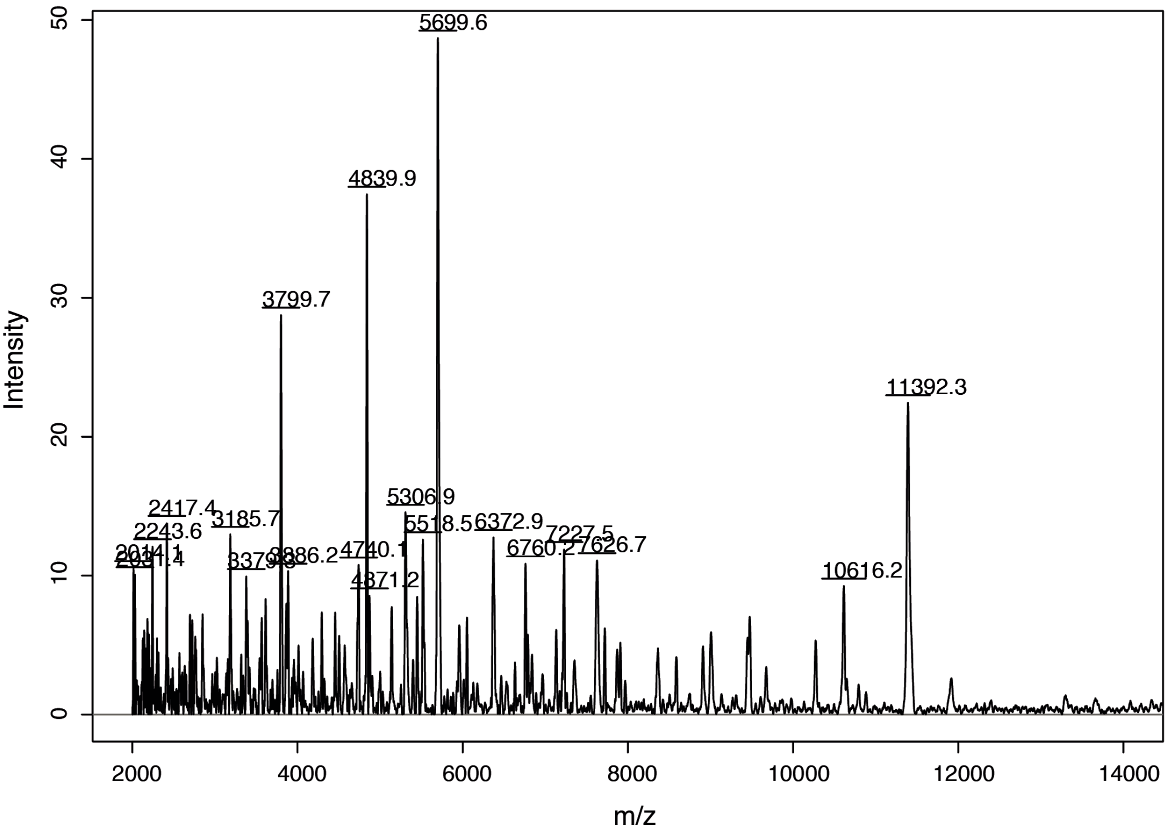

Figure S1: MALDI-TOF MS spectrum of *M. basiliense*. Three MALDI-TOF MS spectra of the *M. basiliense* strain 903442 were imported into R and processed using the packages MALDIQuantForeign and MALDIQuant. The intensities were square root transformed and spectra were smoothed using the SavitzkyGolay algorithm and a half window size of 20. The baselines were subtracted with 20 iterations and the 'SNIP' algorithm, and the three spectra were averaged.

Table S1. Predicted phage regions within the genome of strain 901379. Phage start and end locations are approximate.

Phage start	Phage end	Cargo
3646861	3664310	hypothetical proteins
4111979	4124634	hypothetical proteins
4459037	4468307	hypothetical proteins
4834273	4845140	restriction modification system, hypothetical proteins

Table S2. Predicted pseudogenes within the genome of *M. basiliense* strain 901379.

**Locus tag**	**Putative function**
MB901379_00062	ABC transporter permease
MB901379_00145	conserved oxidoreductase
MB901379_00152	hypothetical protein
MB901379_00177	zinc-type alcohol dehydrogenase AdhD
MB901379_00385	PE-PGRS family protein
MB901379_00888	hypothetical protein
MB901379_01291	short-chain type dehydrogenase/reductase
MB901379_01344	hypothetical protein
MB901379_01600	OsmC-like protein
MB901379_01811	putative membrane protein
MB901379_01914	Nitrite facilitator 1 narK
MB901379_02275	transmembrane serine/threonine-protein kinase
MB901379_02430	Thioredoxin-2
MB901379_02591	putative metal-dependent hydrolase of the TIM-barrel fold protein
MB901379_02817	hypothetical protein
MB901379_02846	hypothetical protein
MB901379_03138	putative metal-dependent hydrolase of the TIM-barrel fold protein
MB901379_03441	iron binding protein
MB901379_04048	NAD(P)/FAD-dependent oxidoreductase

Table S3. Mycobacterial virulence factors identified within the genome of *M. basiliense* strain 901379. Mycobacterial virulence factors listed in VFDB found within strain 901379 are listed. NF: not found.

**Putative virulence factor**		**Encoded by *MB901379_***
**Cell wall**	Antigen 85	*fbpB 02570, fbpC 00247 & 03991, fbpD 04807, fbpA 04808*
	Lipoarabinomannan (LAM)	*pimB 00773*
	MmaA4	*00835*
	phthiocerol dimycocerosat (PDIM)	*01579-01594*
	PcaA	*00679*
**Heat-shock protein**	HspX	*00425 / 03103*
**Iron uptake**	Mycobactin	NF
**Magnesium uptake**	MgtC	*02484*
**Metabolic adaptation**	FadD33	NF
	Isocitrate lyase	*00636 / 02605*
	LipF	*01084 / 02397 / 03285 / 03848*
	Nitrate reductase	*03224*
	PanC/PanD	*04504 / 04503*
**Regulation**	DevRS	*01339-40*
	HspR	*00532 / 02783*
	IdeR	*01824*
	MprAB	*03965-6*
	PhoP	*04329*
	RelA	*01966*

	SigA	*01833*
	SigE	*03677*
	SigF	*01144*
	SigH	*01217*
	WhiB3	*00996*
**Secreted proteins**	ESAT-6/CFP-10	*00456, 02464, 02465, 03236, 03237, 03246, 03247, 03898, 03899, 04527*
**Secretion system**	ESX-1	*01907, 04880*
	ESX-3	NF
	ESX-5	NF
**Stress proteins**	AhpC	*02551*
	KatG	*02595*
	SodA	*04839*
	SodC	*00635*
**Toxin**	Phospholipase C	*01100, 01316, 01946, 03056, 03057, 03182, 03239, 03240, 04130*
**Unclassified**	Erp	*04814*
	HbhA	*00683*
	PE/PE-PGRS	See text

Table S4. Polyketide and non-ribosomal peptide synthesis cluster predictions. Predictions from AntiSmash v 4.0.2 (see methods). NRPS, nonribosomal peptide synthetase; T1pks, Type I polyketide synthase; T3pks, Type 3 polyketide synthase.

**Cluster**	**Type**	**From (bp)**	**To (bp)**	**Notes**
Cluster 1	T1pks	512927	562640	
Cluster 2	T1pks	640901	688160	
Cluster 3	T1pks-NRPS	1148747	1265826	Insertion relative to *M. marinum*
Cluster 4	T1pks-NRPS	1745918	1840006	
Cluster 5	Other	2155376	2198903	
Cluster 6	T3pks	2227768	2268949	
Cluster 7	T1pks-NRPS	2358099	2405576	
Cluster 8	T1pks-NRPS	2407631	2479692	
Cluster 9	T3pks	2579436	2620497	
Cluster 10	NRPS	2934775	2985976	
Cluster 11	T1pks-NRPS	3066759	3175100	Insertion relative to *M. marinum*
Cluster 12	T1pks	3191269	3243775	
Cluster 13	T1pks-NRPS	3551149	3616617	Insertion relative to *M. marinum*
Cluster 14	NRPS (siderophore)	3725896	3788647	
Cluster 15	T3pks (Alkylresorcinol)	4314205	4355251	
Cluster 16	NRPS	4478068	4528702	
Cluster 17	Bacteriocin	4886647	4897453	
Cluster 18	NRPS	4929218	4973657	
Cluster 19	T3pks	5091254	5132468	
Cluster 20	Other	5350407	5394321	
Cluster 21	Lantipeptide	5422007	5447020	
Cluster 22	T1pks	5451753	5497092	
